# The relationship between managed bees and the prevalence of parasites in bumblebees

**DOI:** 10.7717/peerj.522

**Published:** 2014-08-12

**Authors:** Peter Graystock, Dave Goulson, William O.H. Hughes

**Affiliations:** 1 School of Biology, University of Leeds, Leeds, UK; 2 School of Life Sciences, University of Sussex, Brighton, UK

**Keywords:** Pathogen spillover, Pollinator conservation, Commercial bumblebee production, Honeybee

## Abstract

Honey bees and, more recently, bumblebees have been domesticated and are now managed commercially primarily for crop pollination, mixing with wild pollinators during foraging on shared flower resources. There is mounting evidence that managed honey bees or commercially produced bumblebees may affect the health of wild pollinators such as bumblebees by increasing competition for resources and the prevalence of parasites in wild bees. Here we screened 764 bumblebees from around five greenhouses that either used commercially produced bumblebees or did not, as well as bumblebees from 10 colonies placed at two sites either close to or far from a honey bee apiary, for the parasites *Apicystis bombi*, *Crithidia bombi*, *Nosema bombi*, *N. ceranae*, *N. apis* and deformed wing virus. We found that *A. bombi* and *C. bombi* were more prevalent around greenhouses using commercially produced bumblebees, while *C. bombi* was 18% more prevalent in bumblebees at the site near to the honey bee apiary than those at the site far from the apiary. Whilst these results are from only a limited number of sites, they support previous reports of parasite spillover from commercially produced bumblebees to wild bumblebees, and suggest that the impact of stress from competing with managed bees or the vectoring of parasites by them on parasite prevalence in wild bees needs further investigation. It appears increasingly likely that the use of managed bees comes at a cost of increased parasites in wild bumblebees, which is not only a concern for bumblebee conservation, but which may impact other pollinators as well.

## Introduction

In recent years several bumblebee species as well as other pollinators have suffered range declines in parts of Europe, the Americas and Asia (Biesmeijer et al., 2006; Cameron et al., 2011; Goulson, Lye & Darvill, 2008; Potts et al., 2010). Changes in anthropogenic land-use is a major contributing factor to these declines, with agricultural intensification reducing floral diversity and nesting habitats from many pollinators (Goulson et al., 2005; Ricketts et al., 2008; Vanbergen et al., 2013). This has left some bumblebee species fragmented, in small populations with low genetic diversity, something which may make bees more vulnerable to stresses such as parasites (Darvill et al., 2006; Ellis et al., 2006; Evison et al., 2013; Oldroyd, 2007; Whitehorn et al., 2011).

In addition to the stresses of habit loss, pesticide exposure and natural parasites, (Goulson, 2003), the use of managed bees may place additional stresses on bumblebee populations. Honey bees have been managed commercially for crop pollination and honey production for centuries, and are often kept in commercial apiaries with tens to thousands of colonies, substantially increasing the density of bees in an area. Bumblebees are also now commercially produced and used mainly in greenhouses, but also sometimes in polytunnels and open crops, in Europe, North America, South America, New Zealand and Asia to enhance the yields of soft fruit crops (Velthuis & van Doorn, 2006). Although the greenhouses in which commercially produced bumblebees are most commonly used are meant to be closed, the commercially produced bumblebees are frequently found foraging outside the greenhouses, and wild bees have been found foraging inside them (Kraus et al., 2011; Morandin et al., 2001; Murray et al., 2013; Whittington et al., 2004). By freely mixing with wild bumblebees, the deployment of commercially produced bumblebees effectively increases the local density of bumblebees. Bumblebee parasites can be dispersed between bumblebees following shared flower usage (Durrer & Schmid-Hempel, 1994), and, as a result, the rate of parasite transmission between bees will predictably rise with increased pollinator density (Arneberg et al., 1998). In areas utilising commercially produced bumblebees, higher parasite prevalence may be expected to be the result, due to either the spillover of parasites from the commercially produced bumblebees, parasite spillback from wild bumblebees, or stress related to the high pollinator density (Kelly et al., 2009; Power & Mitchell, 2004; Schmid-Hempel, 2011).

The spillover of parasites from one host to another, either intraspecifically or interspecifically, is well known for many organisms (Power & Mitchell, 2004). There is now good evidence that the honey bee parasites *Nosema ceranae* and deformed wing virus have spilled over to bumblebees, with both being virulent and now widespread in their new bumblebee host (Evison et al., 2012; Furst et al., 2014; Genersch et al., 2006; Graystock et al., 2013a; Plischuk et al., 2009). In addition, parasites may also spill over to wild bumblebees from the commercially reared bumblebees used in greenhouses. Colonies of commercially produced bumblebees have been shown in many studies to carry parasites (Colla et al., 2006; Gegear, Otterstatter & Thomson, 2005; Manson, Otterstatter & Thomson, 2010; Meeus et al., 2011; Murray et al., 2013; Otterstatter & Thomson, 2007; Singh et al., 2010; Whittington & Winston, 2003), with the most recent study using sensitive molecular methods finding that three-quarters of the colonies investigated were infected by at least one parasite and confirming that these parasites were in many cases infectious (Graystock et al., 2013b). The introduction of commercially produced bumblebees has been associated with the introduction of foreign parasites and correlated declines in native bumblebee species in Japan, South America and North America, suggesting that the spillover of parasites has occurred on multiple occasions (Arbetman et al., 2012; Colla et al., 2006; Goka et al., 2001; Meeus et al., 2011; Otterstatter & Thomson, 2008; Szabo et al., 2012).

Although attention has focussed on parasite spillover, it is also possible that the use of managed honey bees and commercially produced bumblebees may increase the prevalence of parasites in wild bumblebees via parasite spillback or heightened stress from increased competition when foraging. Managed honey bees or commercially produced bumblebees may become infected with parasites carried by the wild bees, and their unnaturally high density in apiaries or greenhouses may then result in them acting as a reservoir in which the prevalence of parasites becomes high, from which the parasites can then spillback into wild bees (Kelly et al., 2009). The increased competition for resources caused by the introduction of high densities of managed honey bees or commercially produced bumblebees may also stress wild bees due to the increased competition when foraging, which can have negative effects on various fitness components including resistance to parasites (Brown, Loosli & Schmid-Hempel, 2000; Elbgami et al., 2014; Foley et al., 2012; Goulson & Sparrow, 2009; Lafferty & Gerber, 2002; Mallon, Brockmann & Schmid-Hempel, 2003).

The prevalence of parasites in wild bumblebees appears to be greater when the bees are in proximity to greenhouses using commercially produced bumblebee colonies (Colla et al., 2006; Murray et al., 2013; Otterstatter & Thomson, 2008). However, whether this is due to parasite spillover, parasite spillback, or stress, is not always clear. Here we investigate the relationships between commercially reared bumblebees or managed honey bees and the prevalence of a range of parasites in bumblebees. We first examine the relationship between the prevalence of parasites in wild bumblebees and proximity to three farms in which commercially reared bumblebees being used and two greenhouse farms in which they were not being used. In addition, we examine the effect of proximity to honey bees on bumblebee parasite prevalence, using bumblebee colonies located at two sites, either near or far from an apiary.

## Materials and Methods

### The effect of proximity to commercially reared bumblebees

To determine the prevalence of parasites at sites either using commercially produced bumblebees or not, five greenhouse farm sites in England were selected. Sites were selected based on the presence of large scale commercial fruit farms (ca. 50–75 ha) that utilised greenhouses and/or polytunnels for crop growing. Sites were all of comparable size, located in areas of open farmland with no other sites known to be deploying bumblebees within 10 km. Three of the sites in Cambridgeshire, Kent and Essex, were a focal greenhouse in which commercially produced bumblebees were used for the pollination of the greenhouse crops (≈200–300 hives at each site), and two sites in Merseyside and Oxfordshire were a focal greenhouse in which commercially produced bumblebees had not been used (all sites were at least 70 km apart). Bumblebees were collected with a sweep net within 0.5 km of points 0.5, 3 and 5 km from the focal greenhouse sites, with approximately 50 bumblebees collected at each of the three distances for each of the five sites. All bees were collected over a 16 day period in the summer of 2011 (Cambridgeshire, Kent and Essex on 2nd July, 9th July and 11th July respectively, Oxfordshire and Merseyside on 1st and 16th July respectively). A total of 471 bumblebees were collected from around the sites using commercially produced bumblebees (222, 151 and 98 at the Cambridgeshire, Kent and Essex sites respectively) and a total of 293 bumblebees from around the sites not using commercially produced bumblebees (143 and 150 at the Merseyside and Oxfordshire sites respectively). The samples consisted of *B. terrestris*, *B. hortorum*, *B. hypnorum*, *B. lapidarius*, *B. lucorum*, *B. pascuorum* and *B. pratorum*, with most being either *B. terrestris* or *B. lapidarius* (40% and 25% of samples respectively; see Table 1 for detail of the number of each species sampled at each distance at each site). All of these 764 bumblebees were screened for parasites.

**Table 1:** Summary of the bumblebees surveyed. A variety of wild bumblebees were captured to assess the prevalence of parasites in bumblebees sampled at three distances from greenhouses that either were or were not using commercially produced bumblebees. The species, frequency, sex and location of wild bumblebee collected are shown along with their collection site specifics.

Location	Species	*N*	Distance from greenhouses ±0.5 km					
			0.5 km		3 km		5 km	
			F	M	F	M	F	M
**Cambridgeshire** Latitude: 52°18′0.79″N Longitude: 0°3′2.46″W Area: ≈50 acre Number of hives: ≈200^a^	*B. hortoum*	6	3	0	1	0	2	0
	*B. hypnorum*	9	3	0	3	0	3	0
	*B. lapidarius*	99	16	4	43	5	25	6
	*B. lucorum*	33	6	5	4	2	12	4
	*B. pascuorum*	15	5	0	3	0	6	0
	*B. pratorum*	16	0	5	5	2	3	2
	*B. terrestris*	44	15	5	6	0	16	2
**Kent** Latitude: 51°21′13.64″N Longitude: 1°17′8.00″E Area: ≈75 acre Number of hives: ≈300^a^	*B. lapidarius*	21	6	1	5	1	8	0
	*B. lucorum*	12	4	0	3	1	4	0
	*B. pascuorum*	5	3	0	2	0	0	0
	*B. pratorum*	19	8	0	3	1	6	1
	*B. terrestris*	94	26	3	31	3	28	3
**Essex** Latitude: 51°56′0.67″N Longitude: 1°0′18.17″E Area: ≈60 acre Number of hives: ≈240^a^	*B. lapidarius*	30	6	0	5	9	7	3
	*B. lucorum*	2	0	0	2	0	0	0
	*B. pascuorum*	38	16	2	10	1	9	0
	*B. pratorum*	25	21	2	0	1	0	1
	*B. terrestris*	3	3	0	0	0	0	0
**Merseyside** Latitude: 53°30′40.61″N Longitude: 2°47′17.78″W Area: ≈75 acre	*B. hypnorum*	9	2	0	2	0	5	0
	*B. lapidarius*	17	6	0	2	1	8	0
	*B. lucorum*	30	5	0	11	1	11	2
	*B. pascuorum*	2	0	0	0	0	1	1
	*B. pratorum*	12	6	0	2	0	3	1
	*B. terrestris*	73	28	3	23	1	15	3
**Oxfordshire** Latitude: 51°40′10.01″N Longitude: 1°22′38.79″W Area: ≈50 acre	*B. lapidarius*	25	9	0	8	1	6	1
	*B. lucorum*	9	3	0	2	0	4	0
	*B. pascuorum*	12	5	0	4	0	3	0
	*B. pratorum*	16	5	0	2	3	6	0
	*B. terrestris*	88	27	1	29	1	28	2

**Notes.**

a Numbers of hives estimated based on size of farm. As a general rule, producers recommend using 4 bumblebee hives/acre at the beginning of the season, then systematically introducing more hives as the original ones age. The estimates here are based on 4 hives/acre.

### The effect of proximity to managed honey bees

Ten commercially produced *Bombus terrestris audax* bumblebee colonies (Biobest) with 80–100 workers were used to determine the effect of proximity to managed honey bee colonies on parasite prevalence within bumblebee colonies. The colonies were placed on a farm near Tadcaster, West Yorkshire (53°52′N, 1°20′W). Five of the bumblebee colonies were situated on the edge of an agroforestry field containing an apiary with 50, full-size honey bee hives, and the remaining five bumblebee colonies were sited at the edge of a field 1 km away from the apiary, with bees at both locations being in the same landscape with access to similar floral resources (Elbgami et al., 2014). The colonies were placed in a row at the edge of each site, with the same distance between hives in each case. The bumblebee colonies remained at these sites for one month, during which they could forage freely. After this period, 20 bumblebee workers were taken from each colony and screened for the presence of the parasites.

### Molecular screening for parasite presence

A ca. 0.5 cm^3^ sample of midgut, malpighian tubules and fatbody from each bee was homogenised and DNA extracted from the homogenate using 5% Chelex. All DNA samples were amplified for the *18S* Apidae host control gene to confirm the quality of the DNA extraction. Samples were then screened for the presence of the *Apicystis bombi*, *Crithidia bombi*, *Nosema bombi*, *N. ceranae*, *N. apis* and deformed wing virus (DWV) parasites using parasite specific primers and conditions (Chen, Higgins & Feldlaufer, 2005; Gisder & Genersch, 2013; Klee, Tek Tay & Paxton, 2006; Meeus et al., 2010; Table S1). Products were run alongside a size standard on a 1% agarose gel stained with ethidium bromide to confirm amplicon size. Each assay included a negative and a positive control.

### Statistical analysis

The prevalence and richness of parasites was compared between sites in which greenhouses did or did not use commercially produced bumblebees, and between the sites near to or far from the honey bee apiary using generalized linear models (GLM) with the likelihood ratio *χ*^2^ statistic. The parasite richness (number of parasite species detected in a single host) was compared between sites using a negative binomial distribution and log link function and changes in the prevalence of individual parasites with a binomial distribution and logit link function. When looking at the effect of commercially produced bumblebees, site type (greenhouses in which commercially produced bumblebees were or were not used), transect distance, and site location nested within site type were included as factors, with the species and sex of bumblebees sampled also included as factors. We did not include sampling dates in these models because it covaried with site, but checked for temporal autocorrelation using Box–Ljung tests and retested the GLM without site and instead including sampling date (number of days after the first sample was collected) as a covariate. We checked for spatial autocorrelation using Moran’s I (Rogerson, 2010). When looking at the effect of managed honey bees, location (near to or far from the apiary), and colony nested within location, were used as factors. Nonsignificant terms were removed stepwise based on log-likelihood ratio tests in all cases to obtain the minimum adequate models (Table S2). All analyses were carried out in PASW Statistics 20 (IBM, Armonk, NY, USA).

## Results

### The effect of commercially produced bumblebees on parasite prevalence in wild bumblebees

**Figure 1:**
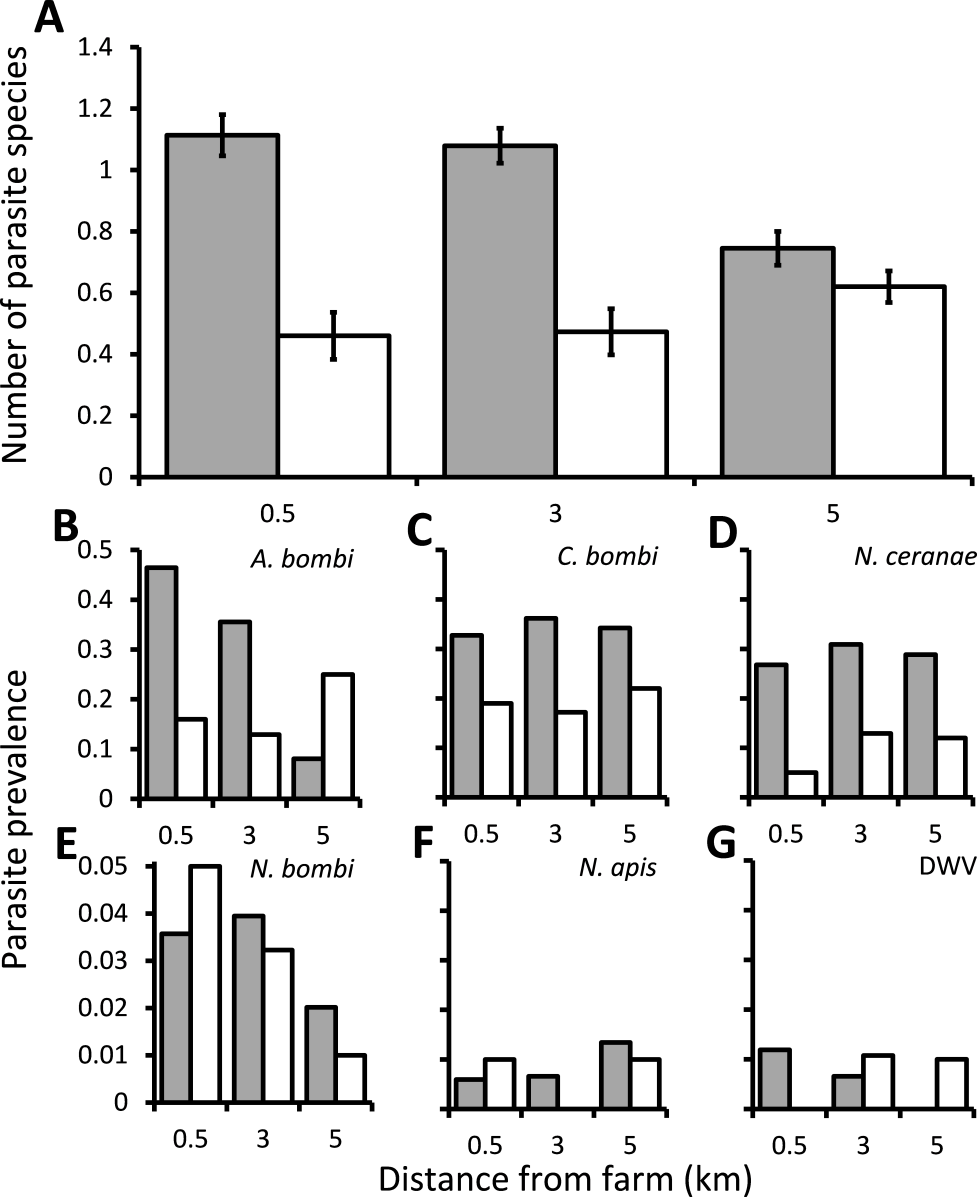
The effect of commercially produced bumblebees on parasite prevalence. Prevalence of parasites in bumblebees sampled within 0.5 km of locations that were 0.5, 3 or 5 km from greenhouses that were either using (grey columns) or not using (white columns) commercially produced bumblebee colonies. (A) The mean ± s.e. parasite richness (number of species) infecting individual bees. (B–G) The proportion of bumblebees sampled which were positive for the *A. bombi*, *C. bombi*, *N. ceranae*, *N. bombi*, *N. apis* and deformed wing virus (DWV) parasites.

Overall, most wild bumblebees had either no infections (40.7%) or infection by a single parasite species (40.3%), with cases of bumblebees infected by two or three parasite species being rare (16.8% and 2.1% respectively). There was a significant interaction between the distance from the greenhouses and whether they were or were not using commercially produced bumblebees on the numbers of parasite species that infected bumblebees (*χ*^2^ = 6.78, d.f. = 2, *P* = 0.034), and this was not affected by either the species or sex of the bumblebee (*χ*^2^ = 3.04, d.f. = 6, *P* = 0.804 and *χ*^2^ = 0.874, d.f. = 1, *P* = 0.35, respectively). The numbers of parasite species recorded decreased with distance from the greenhouses at sites which were using commercially produced bumblebees, but were unaffected by distance at the sites which were not using these bees (Fig. 1A). There was also a significant difference between individual sites nested within categories of using or not using commercially produced bumblebees (*χ*^2^ = 29.0, d.f. = 3, *P* < 0.001), but the bees sampled from around sites using commercially produced bumblebees nevertheless had significantly more parasite species overall when controlling for this (*χ*^2^ = 23.2, d.f. = 1, *P* < 0.001). The samples were collected within a relatively short 16 day period and showed no temporal autocorrelation (Box–Ljung tests all *P* > 0.05), with there being no effect of sampling date on the number of parasite species found (*χ*^2^ = 2.86, d.f. = 1, *P* = 0.091). There was also no spatial autocorrelation (Moran’s *I* = 0.062; a value of 1 indicates perfect correlation and of 0 indicates random dispersion). Of the individual parasites, the prevalence of both *A. bombi* and *N. ceranae* were affected significantly by the interaction between distance and whether sites were using commercially produced bumblebees or not (*χ*^2^ = 44.5, d.f. = 2, *P* < 0.001, and *χ*^2^ = 7.98, d.f. = 2, *P* = 0.019, respectively). *A. bombi* was more commonly close to greenhouses when those greenhouses were using commercially produced bumblebees but showing little effect of distance when they were not (Fig. 1B). *N. ceranae*, in contrast, increased with distance from the greenhouses at the sites not using commercially produced bumblebees but was little affected by distance at the sites where they were (Fig. 1D). *Crithidia bombi* was more prevalent in bumblebees caught from around sites using commercially produced bumblebees than those not using them (*χ*^2^ = 15.1, d.f. = 1, *P* < 0.001) but displayed no proximity effect (*χ*^2^ = 0.756, d.f. = 2, *P* = 0.685; Fig. 1C). *N. ceranae* was the only parasite to show a significant effect of the species or sex of bumblebee sampled (Table S2), which was due to all 7 of the *B. hortorum* bees sampled being workers that were infected by *N. ceranae*. The prevalence of *N. bombi, N. apis* and DWV in bumblebees caught were all under 1% and were not affected by any variables (Table S2; Fig. 1).

### The effect of managed honey bees on parasite prevalence within bumblebee colonies

**Figure 2:**
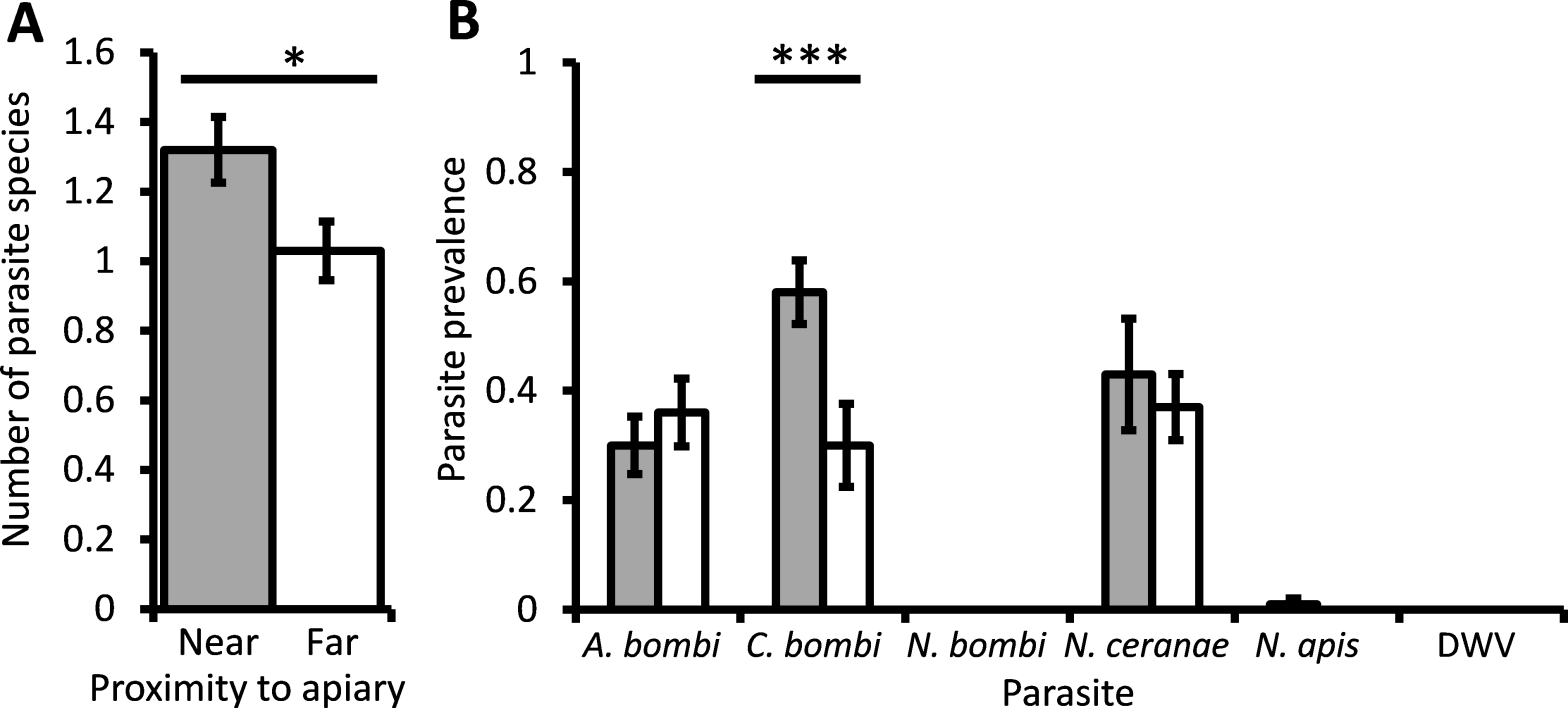
The effect of managed honeybees on parasite prevalence. The mean ± s.e. parasite richness (number of species) per bumblebee (A), and the prevalence of six parasites per bumblebee colony (B), that were located either at a site near to (grey columns) or 1 km away from (white columns) a honey bee apiary. Asterisks indicate columns for which there was a significant difference between colonies located near to and far from the apiary (* *P* < 0.05; *** *P* < 0.001).

The mean parasite richness varied between bumblebee colonies but was significantly higher overall in colonies located in close proximity to honey bees (*χ*^2^ = 5.66, d.f. = 1, *P* = 0.017; Fig. 2A). The average prevalence of *C. bombi* in bumblebee colonies near honey bees was 58%; significantly higher than the 30% found in colonies far from honey bees (*χ*^2^ = 17.9, d.f. = 1, *P* < 0.001; Fig. 2B). The prevalence of *A. bombi* and *N. ceranae* in colonies located near honey bees averaged 30% and 43%, respectively, which did not differ from the prevalence of these parasites in colonies far from honey bees (*χ*^2^ = 0.83, d.f. = 1, *P* = 0.36; *χ*^2^ = 0.27, d.f. = 1, *P* = 0.61). *N. ceranae* prevalence did, however, differ between colonies within sampling sites (*χ*^2^ = 25.07, d.f. = 8, *P* = 0.002). *N. apis* was only found in bumblebee colonies located near to honey bee hives, but had a very low prevalence and thus did not differ significantly between the sites (*χ*^2^ < 0.01, d.f. = 1, *P* = 0.993). *Nosema bombi* and DWV were not detected in any of the 200 bumblebees sampled.

## Discussion

Although the study involved only a very limited number of sites and must thus be interpreted with caution, the results suggest that the prevalence of parasites in bumblebees may be affected by the presence of managed bees. The prevalence of *A. bombi* and *C. bombi* was respectively 12% and 15% higher in bumblebees near greenhouses at the three sites using commercially produced bumblebees compared to the two sites not using these bees, and the prevalence of *Apicystis bombi* was also much higher 0.5 km from the greenhouses compared with 5 km away from them. The samples were collected during a relatively short 16 day period and the differences between sites were not due to spatial or temporal autocorrelation. Bumblebees in colonies located close to the managed honey bee apiary had higher levels of the parasite *C. bombi* compared to bumblebees in colonies that were located 1 km away from the apiary. Although data from more sites are obviously needed to draw firm conclusions, the results suggest that the presence of managed colonies of either bumblebees or honey bees may increase the prevalence of parasites in wild bumblebees.

A wide diversity of parasites were detected in the wild bumblebees collected near greenhouses, including the bumblebee parasites *A. bombi*, *C. bombi* and *N. bombi*, and the honey bee parasites *N. ceranae*, *N. apis* and DWV, all of which have also been identified in commercially produced bumblebees (Graystock et al., 2013b). *N. bombi*, *N. apis* and DWV were very rare (<1% prevalence) but the other parasites were more common. In general, the parasite richness within wild bumblebees increased with proximity to greenhouses utilising commercially produced bumblebees and bumblebees caught from around such greenhouses had a higher prevalence of *A. bombi* and *C. bombi* than those caught around greenhouses not using commercially reared bumblebees. Whether through parasite spillover, parasite spillback, or the stress of increased competition, commercially produced bumblebees appear to be increasing the prevalence of parasites in local bumblebees. These findings support previous, microscopy-based studies that found a higher prevalence of parasites near sites using commercially produced bumblebees (Colla et al., 2006; Murray et al., 2013; Otterstatter & Thomson, 2008). The effect of greenhouses using commercially produced bumblebees on the prevalence *A. bombi* appeared to be influenced by proximity to the focal glasshouse site. This perhaps suggests either a recent introduction from the greenhouses or that the dispersal of the parasite through the environment is relatively limited. There have been no studies of the horizontal transmission of *A. bombi*, although it has been commonly found at a low prevalence when bees are examined using less sensitive microscopy methods (Goulson, Whitehorn & Fowley, 2012; Shykoff & Schmid-Hempel, 1991). Worryingly this parasite has been implicated in bumblebee declines in South America (Arbetman et al., 2012). *Crithidia bombi* was also found to be more prevalent at sites using commercially produced bumblebees. Unlike *A. bombi*, there was no proximity effect found, but *C. bombi* is known to readily transmit between bumblebees and may therefore disperse rapidly through the environment (Durrer & Schmid-Hempel, 1994). The prevalence of none of the other parasites investigated differed between sites with or without commercially produced bumblebees. *Nosema ceranae* was abundant at some sites but completely absent at other sites. *Nosema ceranae*, is an emergent honey bee parasite that is implicated in the collapse of honey bee colonies in some, but not all, areas (Fries, 2010; Higes et al., 2008; Klee et al., 2007; Paxton, 2010; Paxton et al., 2008; Roberts & Hughes, 2014), and which has been shown to be widespread and virulent in bumblebees (Furst et al., 2014; Graystock et al., 2013a; Plischuk et al., 2009).

Although based on only two sites, the comparison of the site with honey bee hives and the site 1 km from the hives suggested that proximity to managed honey bee colonies may also have an effect on parasite prevalence in bumblebee colonies. Although the levels of *N. bombi*, *N. apis* and DWV were too low for any conclusions, and *A. bombi* and *N. ceranae* did not differ between the two sites, *C. bombi* was significantly more prevalent in bumblebee colonies that were near to the honey bee hives. This effect could not be due to spillover, because *C. bombi* is unable to infect honey bees (Ruiz-González & Brown, 2006). It could, however, be due to stress from competition leading to the bumblebees close to the honey bee apiary being more susceptible to infection (Brown, Loosli & Schmid-Hempel, 2000; Elbgami et al., 2014; Goulson & Sparrow, 2009; Lafferty & Gerber, 2002; Mallon, Brockmann & Schmid-Hempel, 2003), or to the honey bees vectoring *C. bombi*. The potential role of stress and parasite spillback in driving elevated parasite prevalence in wild pollinators has been largely ignored and would warrant further investigation.

Our results suggest that managed colonies of bees may increase the prevalence of parasites in bumblebees. The results here are based on only very few sites and clearly further studies are needed using far more sites to establish their generality. It will be important for such studies to consider the potential for parasite spillback and stress-related effects, in addition to parasite spillover. It is clear that as long as there is mixing between managed and wild bees, there is the potential for wild populations to be at risk from the effects on host-parasite dynamics. These effects could prove to be a major conservation threat to bumblebees.

## Supplemental Information

10.7717/peerj.522/supp-1 Table S1 PCR mixes and conditions for the detection of the various parasites

10.7717/peerj.522/supp-2 Table S2 Statistical models Statistical models used to analyze the richness and prevalence of parasites in bumblebees sampled either 0.5, 3 or 5 km (±0.5 km) from three farms using commercially produced bumblebee colonies and two farms not using commercially produced bumblebees.
