# Supplementary material for: The relationship between managed bees and the prevalence of parasites in bumblebees
Source: PeerJ. 2014 Aug 12;2:e522. doi: 10.7717/peerj.522 (PMC4137657; doi:10.7717/peerj.522)
Supplement: Table S1 [file peerj-02-522-s001.docx]

| **Primers & source** | **Assay mix** | | | | | | | | **Thermal cycling** | | | **Amplicon size (bp)** |
| --- | --- | --- | --- | --- | --- | --- | --- | --- | --- | --- | --- | --- |
|  | **dNTP (nM)** | **MgCl_2_ (nM)** | **5xbuffer (μl)** | **Taq (U)** | **Primer F (μM)** | **Primer R (μM)** | **Template (μl)** | **Total volume (μl)** | **1**  **Denaturing**  **Min \| Temp** | **2 Replication Sec \| Temp** | **3**  **Elongation Min \| Temp** |  |
| *Nosema bombi*  (Klee et al. 2006) | 0.3 | 3.75 | 2 | 0.25 | 0.2 | 0.2 | 2 | 10 | 4 \| 95 | 35x  60 \| 95  60 \| 50  60 \| 72 | 4 \| 72 | 323 |
| **Nosema apis*^Na^ & *N. ceranae*^Nc^  (Gisder & Genersch 2013) | 0.2 | 1.5 | 2 | 2.5 | 0.2^Na^  0.2^Nc^ | 0.2^Na^  0.2^Nc^ | 1 | 10 | 4\| 95 | 35x  60 \| 94  60 \| 58  60 \| 72 | 5 \| 72 | 297^Na^  662^Nc^ |
| *Apicystis bombi*  (Meeus et al. 2010) | 0.4 | 1.5 | 2 | 1.25 | 0.5 | 0.5 | 1 | 10 | 2 \| 94 | 35x  30 \| 94  30 \| 60  45 \| 72 | 3 \| 72 | 260 |
| *Apidae*^A^ (internal) and *Crithida bombi*^Cb^  (Meeus et al. 2010) | 0.4 | 1.5 | 3 | 1.25 | 0.1^A^  0.5^Cb^ | 0.2^A^  0.5^Cb^ | 2 | 15 | 2 \| 94 | 35x  30 \| 94  30 \| 57  45 \| 72 | 3 \| 72 | 130^A^  420^Cb^ |
|  |  |  |  |  |  |  |  |  |  |  |  |  |
| **RT-PCR** | **Probe (nM)** | **Taqman Fast Virus 1-step Master mix(μl)** | | | **Primer F (μM)** | **Primer R (μM)** | **Template (μl)** | **Total volume (μl)** | **1**  **Reverse transcription**  **Min \| Temp** | **2**  **Denaturing**  **Sec \| Temp** | **3**  **Annealing and elongation Time \| Temp** | **Amplicon size (bp)** |
| Deformed wing virus  (Chen et al. 2005) | 200 | 5 | | | 0.65 | 0.65 | 2 | 10 | 5 \| 50 | 20 \| 95 | 40x  3 s \| 95  3 min \| 60 | 702 |
