# Supplementary material for: The relationship between managed bees and the prevalence of parasites in bumblebees
Source: PeerJ. 2014 Aug 12;2:e522. doi: 10.7717/peerj.522 (PMC4137657; doi:10.7717/peerj.522)
Supplement: Table S2 — Statistical models used to analyze the richness and prevalence of parasites in bumblebees sampled either 0.5, 3 or 5 km (±0.5 km) from three farms using commercially produced bumblebee colonies and two farms not using commercially produced bumblebees. [file peerj-02-522-s002.docx]

| **TERM IN MODEL** | ***χ^2^*** | **df** | ***P*** |
| --- | --- | --- | --- |
| **Overall number of parasite species:** |  |  |  |
| Commercial bumblebees presence | 23.2 | 1 | <0.001 |
| Site nested within status | 29.0 | 2 | <0.001 |
| Distance from greenhouses | 0.112 | 3 | 0.946 |
| Species of bumblebee sampled | 3.04 | 6 | 0.804 |
| Sex of bumblebee sampled | 0.874 | 1 | 0.35 |
| Commercial bumblebees presence*Distance from greenhouses | 6.78 | 2 | 0.034 |
| ***Apicystis bombi:*** |  |  |  |
| Commercial bumblebees presence | 1.92 | 1 | 0.166 |
| Site nested within status | 113.1 | 3 | <0.001 |
| Distance from greenhouses | 14.1 | 2 | 0.001 |
| Species of bumblebee sampled | 6.59 | 6 | 0.36 |
| Sex of bumblebee sampled | 2.05 | 1 | 0.152 |
| Commercial bumblebees presence*Distance from greenhouses | 44.5 | 2 | <0.001 |
| ***Crithidia bombi:*** |  |  |  |
| Commercial bumblebees presence | 15.1 | 1 | <0.001 |
| Site nested within status | 2.79 | 3 | 0.425 |
| Distance from greenhouses | 0.315 | 2 | 0.854 |
| Species of bumblebee sampled | 1.72 | 6 | 0.943 |
| Sex of bumblebee sampled | 0.019 | 1 | 0.89 |
| Commercial bumblebees presence*Distance from greenhouses | 0.756 | 2 | 0.685 |
| ***Nosema ceranae:*** |  |  |  |
| Commercial bumblebees presence | 0.009 | 1 | 0.994 |
| Site nested within status | 146.1 | 3 | 0.104 |
| Distance from greenhouses | 4.53 | 2 | <0.001 |
| Species of bumblebee sampled | 27.4 | 6 | <0.001 |
| Sex of bumblebee sampled | 7.64 | 1 | 0.006 |
| Commercial bumblebees presence*Distance from greenhouses | 7.98 | 2 | 0.019 |
| ***Nosema bombi:*** |  |  |  |
| Commercial bumblebees presence | 0.068 | 1 | 0.794 |
| Site nested within status | 1.45 | 3 | 0.694 |
| Distance from greenhouses | 3.41 | 2 | 0.182 |
| Species of bumblebee sampled | 4.72 | 6 | 0.58 |
| Sex of bumblebee sampled | 0.034 | 1 | 0.854 |
| Commercial bumblebees presence*Distance from greenhouses | 0.626 | 2 | 0.731 |
| ***Nosema apis:*** |  |  |  |
| Commercial bumblebees presence | 0.009 | 1 | 0.994 |
| Site nested within status | 4.07 | 3 | 0.254 |
| Distance from greenhouses | 2.01 | 2 | 0.366 |
| Species of bumblebee sampled | 4.83 | 6 | 0.566 |
| Sex of bumblebee sampled | 0.245 | 1 | 0.62 |
| Commercial bumblebees presence*Distance from greenhouses | 0.935 | 2 | 0.626 |
| **deformed wing virus:** |  |  |  |
| Commercial bumblebees presence | 0.001 | 1 | 0.994 |
| Site nested within status | 5.65 | 3 | 0.13 |
| Distance from greenhouses | 1.32 | 2 | 0.516 |
| Species of bumblebee sampled | 3.69 | 6 | 0.718 |
| Sex of bumblebee sampled | 1.53 | 1 | 0.216 |
| Commercial bumblebees presence*Distance from greenhouses | 4.87 | 2 | 0.088 |
